# RNA‐Seq of Cultured Peripheral Blood Lymphocytes Improves Identification of Cryptic Splicing Defects in Rare Disease Diagnostics

**DOI:** 10.1155/humu/9635551

**Published:** 2026-01-08

**Authors:** Jinlin Ren, Congling Dai, Fei Meng, Pan Zhang, Chunbo Xie, Wenjuan Xiao, Wenbin He, Shimin Yuan, Xiurong Li, Qianjun Zhang, Weiling Tang, Liang Hu, Zixu Chen, Guangxiu Lu, Juan Du, Sicong Zeng, Ge Lin

**Affiliations:** ^1^ Hunan Guangxiu Hospital, Hunan Guangxiu Hi-tech Life Technology Co., Ltd, Hunan Normal University, Changsha, Hunan, China, hunnu.edu.cn; ^2^ Clinical Research Center for Reproduction and Genetics in Hunan Province, Reproductive and Genetic Hospital of Citic-Xiangya, Changsha, Hunan, China, zxxyyy.cn; ^3^ Institute of Reproduction and Stem Cell Engineering, School of Basic Medical Science, Central South University, Changsha, China, csu.edu.cn; ^4^ National Engineering and Research Center of Human Stem Cells, Changsha, China; ^5^ Hunan International Scientific and Technological Cooperation Base of Development and carcinogenesis, Changsha, Hunan, China

**Keywords:** peripheral blood, phytohemagglutinin-activated peripheral blood cells (PHACs), preconception genetic counseling, rare diseases, RNA sequencing (RNA-seq)

## Abstract

Accurate identification of the genetic determinants of rare diseases is essential for effective recurrence‐risk management and informed reproductive decision‐making. Although whole‐exome sequencing (WES) and whole‐genome sequencing (WGS) have significantly improved diagnostic capabilities, a subset of affected families still receives no definitive molecular diagnosis. RNA sequencing (RNA‐seq) has emerged as a promising complementary diagnostic tool, yet its clinical implementation in the context of preconception genetic counseling remains underexplored. We used phytohemagglutinin‐activated peripheral blood cells (PHACs) as a robust RNA source and enhanced conventional RNA‐seq through the integration of three analytical innovations: (1) transcript isoform distribution (TID) analysis, (2) realignment against the MANE (Matched Annotation from NCBI and EMBL‐EBI) reference transcriptome, and (3) pharmacological induction–based cryptic splicing detection. This optimized pipeline was applied to 55 rare‐disease families with negative WES/WGS results who were undergoing preconception genetic counseling. Based on prior evaluations, families were grouped as VUS (*n* = 7), suspected‐gene/variant‐negative (*n* = 10), and unsolved/no‐candidate (*n* = 38). PHACs showed reduced interindividual variability and higher RNA integrity than fresh PBMCs (median RIN: 9.77 vs. 8.97; *p* < 0.0001). The optimized workflow improved diagnostic yield by 2.2‐fold (20% vs. 9%). Stratified analysis revealed positive rates of 71% (VUS), 40% (suspected‐gene/variant‐negative), and 5.2% (unsolved/no‐candidate). Among the 11 positive cases, 10 received definitive diagnoses, leading to diverse reproductive decisions. This enhanced RNA‐seq workflow provides a clinically applicable and scalable strategy for improving molecular diagnostics in reproductive and preconception settings, offering a valuable model for future clinical transcriptomics.

## 1. Introduction

In preconception genetic counseling, couples with a personal or family history of rare disorders seek informed reproductive decisions, often guided by comprehensive molecular diagnoses. To mitigate the risk of transmitting pathogenic variants, interventions such as prenatal testing, preimplantation genetic testing (PGT), or the use of donor gametes are considered. Whole‐exome sequencing and whole‐genome sequencing (WES/WGS) have become the mainstay of rare disease diagnostics, identifying causal variants in approximately 28.8%–55% of cases [1, 2]. However, a proportion of cases remain unresolved, necessitating multiomics integration for comprehensive diagnostic insights. As the intermediary between DNA and protein, RNA plays a pivotal role in elucidating disease mechanisms, with an estimated 9%–30% of noncoding mutations contributing to pathology through disrupted RNA processing and expression [3, 4]. RNA sequencing (RNA‐seq) demonstrates substantial potential to improve diagnostic accuracy and reveal novel disease mechanisms—particularly for rare disorders where DNA analysis alone fails to yield clear diagnoses—by enabling transcriptome‐wide aberration detection in disease‐relevant genes [5–7]. Its diagnostic utility has been validated across diverse patient‐derived tissues, including muscle biopsies [8, 9], peripheral blood mononuclear cells [10], skin fibroblasts [11], transdifferentiated fibroblasts [12], and B‐lymphoblastoid cell lines [13].

Disease‐affected tissues represent the optimal resource for RNA‐seq analysis due to the tissue‐specific nature of gene expression [8, 11, 14]. However, clinical testing is often limited to readily accessible samples such as blood. Notably, studies on rare disease cohorts have demonstrated that blood‐derived RNA expression profiles encompass approximately 3000 Mendelian genes [15], effectively capturing disease‐associated transcripts across neurological, musculoskeletal, hematological, and ophthalmological disorders [10]. Detection of aberrant mRNA splicing in blood samples has been shown to increase molecular diagnostic yields beyond genome sequencing alone [16, 17]. With the increasing adoption of telemedicine in preconception genetic counseling, peripheral blood samples have become the most practical specimen type due to their ease of collection. However, their inherent variability in cellular composition and susceptibility to degradation during transport pose significant challenges for RNA‐seq applications. For genetic laboratories, particularly those specializing in reproductive healthcare, optimizing peripheral blood sample preparation is essential to ensure reliable and clinically actionable RNA‐seq results [18].

In conventional RNA‐seq analysis, after aligning the quality‐filtered reads to the reference genome, outliers in gene expression, splicing, and allelic expression imbalance (AEI) are analyzed [6, 19]. Abnormal splicing events are typically identified as outliers only when they generate novel isoforms that are not cataloged in existing databases. However, not all transcripts documented in these databases are functionally active. The pervasive presence of multiple transcript isoforms derived from a single gene locus may confound the identification of aberrant splicing events in functionally relevant transcripts. Moreover, due to the discrepancies between the human transcriptome and the reference genome, mapping RNA‐seq reads originating from transcripts onto the genome presents significant challenges. Reads spanning only a few junctional bases can be misaligned to adjacent introns or incorrect genomic loci, potentially resulting in false negatives during read mapping [20]. Furthermore, intrinsic cellular surveillance mechanisms, most notably nonsense‐mediated mRNA decay (NMD), actively degrade transcripts containing premature termination codons, thereby masking aberrant splicing events from detection [21].

To address the aforementioned limitations of conventional RNA‐seq, we developed an integrated framework utilizing phytohemagglutinin‐activated peripheral blood cells (PHACs) in combination with three synergistic analytical innovations: (1) transcript isoform distribution (TID) analysis, (2) read realignment against the Matched Annotation from NCBI and EMBL‐EBI (MANE)–selected reference transcriptome, and (3) pharmacological induction–based cryptic splicing detection. In a cohort of 55 families undergoing preconception genetic counseling with unresolved diagnoses after WES/WGS analysis, this approach increases the positive detection rate from 9% (5/55) to 20% (11/55) compared to the conventional whole‐blood RNA‐seq workflow. Ultimately, 10 families received definitive molecular diagnoses, enabling informed reproductive decisions for their subsequent pregnancies.

## 2. Materials and Methods

### 2.1. Subjects

This ethically approved study (LL‐SC‐2022‐005) was conducted at the Reproductive and Genetic Hospital of CITIC‐Xiangya. We prospectively enrolled 55 preconception genetic counseling families (124 individuals) with suspected monogenic disorders, all seeking molecular diagnoses, to guide assisted reproductive interventions. All families had unresolved diagnoses after clinical‐grade whole‐exome sequencing (WES) reanalysis (*n* = 51) or whole‐genome sequencing (WGS) (*n* = 4). Eleven ethnically matched controls undergoing in vitro fertilization (IVF) for nongenetic indications (e.g., tubal/obstructive infertility) were concurrently enrolled, with rigorous exclusion of any inherited disease histories. Comprehensive biospecimen information and clinical annotations are cataloged in Tables S1 and S2.

### 2.2. Peripheral Blood Mononuclear Cell (PBMC) and PHAC Acquisition

Peripheral blood samples were collected in two forms: fresh peripheral blood and blood for lymphocyte culture.

For fresh peripheral blood, a total of 19 samples (2 mL each) were collected on‐site using EDTA‐K2 as an anticoagulant. Within 4 h of collection, PBMCs were isolated using a lymphocyte isolation kit (LTS10771; TBD Science, China) following the manufacturer′s instructions. The isolated PBMCs were directly used for subsequent RNA extraction and flow cytometry analysis.

For lymphocyte culture, a total of 126 peripheral blood samples were collected, including 60 on‐site samples and 66 samples transported under refrigeration (4°C) to the laboratory, approximately 3 days. Each blood sample (2 mL) was anticoagulated with heparin. Blood samples were cultured with phytohemagglutinin (PHA) either immediately or within 4 days after collection. Sterile tubes containing 1 mL of RPMI 1640 medium (pH 7.2–7.4) (R8758; Sigma‐Aldrich, United States), 10% (*v*/*v*) fetal calf serum (F8318; Sigma‐Aldrich, United States), 1% (*v*/*v*) penicillin/streptomycin (CL12331; ChemeGen, China), and 100 *μ*g/mL PHA (9008‐97‐3; MedChemExpress, United States) were prepared. Then, 400 *μ*L of heparinized blood from each sample was added to the tube and incubated at 37°C with 5% CO_2_ for 72 h. For extended cultivation in PHACs, cycloheximide (CHX) (239765; Sigma‐Aldrich, United States) or mitomycin C (MMC) (M5353; Sigma‐Aldrich, United States) was added at concentrations of 80 *μ*g/mL and 0.16 ng/*μ*L, respectively, during the last 16–18 h. After cultivation, the blood–medium mixture was transferred to a 15‐mL centrifuge tube. Red blood cells were lysed using red blood cell lysis buffer (158904; QIAGEN, Germany) by incubating on ice for 15 min, followed by centrifugation at 2000 rpm at 4°C for 5 min. The supernatant was discarded, and the pellet was resuspended in 3 mL of red blood cell lysis buffer, incubated on ice for 15 min, and centrifuged again under the same conditions. The resulting pellet was resuspended in 5 mL of precooled PBS and centrifuged at 2000 rpm at 4°C for 5 min. The supernatant was discarded, and the lymphocytes were divided into three portions: the first for subsequent RNA extraction, the second for flow cytometry analysis, and the third resuspended in 1 mL of T lymphocyte storage solution (IMC‐705; IMMO BIOTECH, China) and stored in liquid nitrogen for potential future drug treatment after revival.

### 2.3. Flow Cytometry Analysis

Cells were dissociated into single‐cell suspensions and resuspended in FACS buffer (1% FBS in PBS) at a concentration of 1 × 10^6^ cells per 100 *μ*L. Following fixation with 4% paraformaldehyde (PFA) and washing with 1% FBS buffer, cells were stained with the following fluorochrome‐conjugated antibodies (BD Biosciences, United States): anti‐CD45, anti‐CD3, anti‐CD19, anti‐CD8, and anti‐CD4. After 30‐min incubation at 4°C in the dark, cells were washed twice with FBS buffer and resuspended in fresh FACS buffer for analysis. Flow cytometry and data analysis were performed using an Accuri C6 flow cytometer (BD Biosciences).

### 2.4. RNA Preparation and RNA‐Seq

TRIzol Reagent (Cat. No. 15596026; Invitrogen, United States) was employed to isolate total RNA following the manufacturer′s protocol. RNA quality was evaluated via RNA integrity number (RIN) and RNA quality number (RQN) determination using an Agilent 2100 Bioanalyzer. All samples met the predefined quantity and quality criteria, with total RNA amounts ≥ 250 ng and RIN/RQN values ≥ 7.

Library preparation for transcriptome sequencing was conducted as follows: mRNA was purified from total RNA using Poly‐T oligo‐conjugated magnetic beads. After RNA fragmentation, first‐strand cDNA was synthesized with random hexamer primers, followed by second‐strand cDNA synthesis. The library was constructed subsequent to end repair, A‐tailing, adapter ligation, size selection, amplification, and purification.

Following library quality control, clustering and sequencing were performed. Specifically, different libraries were pooled according to their effective concentrations and targeted data volumes. The 5 ^′^‐end of each library was phosphorylated and cyclized, followed by loop amplification to generate DNA nanoballs (DNBs). These DNBs were finally loaded onto a flow cell for sequencing on the DNBSEQ‐T7 platform.

Sequencing was conducted at BGI. Nonstranded mRNA was selected via Poly‐A enrichment (for DNBSEQ library construction), followed by paired‐end 150 bp sequencing on the MGISEQ‐T7 platform, achieving a sequencing depth of ≥ 80 million reads per sample.

### 2.5. Gene Expression Analysis

We used the fastp (v0.20.0) software package [22] to filter out low‐quality bases from the raw sequencing reads and to trim adapter sequences introduced during library preparation. Filtered reads were then aligned to the human reference genome GRCh38 (Ensembl database Version GRCh38 release 98 gene annotation) using STAR (v2.7.3a) software [23], producing BAM files of the alignment results. The expression levels of the genes were quantified using StringTie (v2.0.4) [24] in counts and transcripts per million (TPM). Differentially expressed genes were analyzed using the R package DESeq2 [25] (filtered with *q* − value ≤ 0.01 and fold change ≥ 2). Abnormally expressed genes were analyzed in each sample using the R package OUTRIDER [26]. To assess gene expression across all samples, we first filtered our gene list to include only protein‐coding genes. Expressed protein‐coding genes were then identified using a cut‐off of mean TPM > 1 and mean coverage > 10×.

### 2.6. OMIM Gene Expression in PBMCs and PHACs

The clinical diagnosis of Mendelian disorders typically relies on a recognizable pattern of phenotypic features associated with pathogenic variants in single genes [13]. Therefore, clinicians and laboratory directors must understand phenotype–gene information during testing. To compare RNA‐seq‐captured phenotype‐associated gene profiles between PBMCs and PHACs, we set an expressed gene threshold of median TPM > 1 and median coverage > 10× across exonic regions to filter expressed protein‐coding genes. A curated list of Mendelian genes across nine disease categories (downloaded from publicly available disease‐specific panels; see Table S4; (Figure 1A, B) was annotated, and the proportion of genes that meet the analysis thresholds was compared between two sample types (PBMCs and PHACs) within these nine disease‐associated gene sets.

Figure 1Comparison of RNA information acquisition between PHACs and PBMCs and across disease types. (a) Number of OMIM genes detected in PBMCs and PHACs. The cutoff for detection was set at a median TPM > 1 and median coverage > 10×. (b) Comparison of the proportion of detectable disease catalog genes in PBMCs and PHACs (*p* value ≤ 0.05, *t*‐test). (c) Distribution of disease types and clinical stratification in the cohort.

**Figure:**
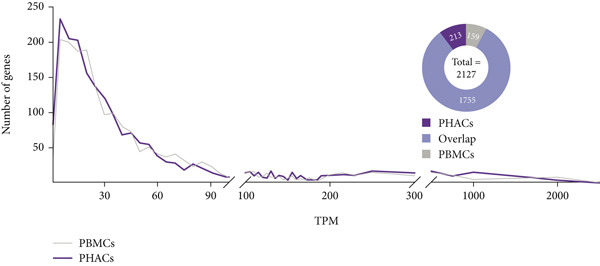
(a)

**Figure:**
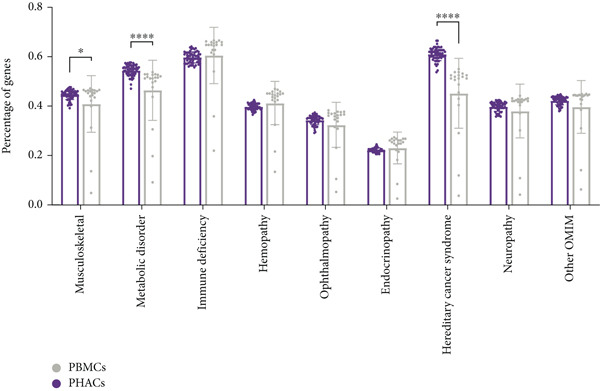
(b)

**Figure:**
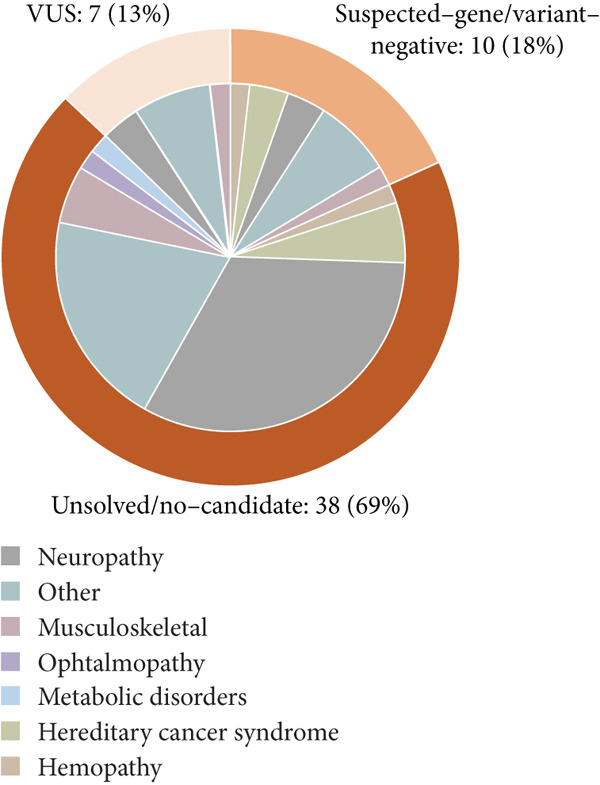
(c)

### 2.7. Conventional Outlier Analysis in Expression, Splicing, and AEI

Previous large‐sample studies provided a logical framework for the clinical analysis of RNA‐seq data [10]. Based on genomic structure and transcriptome data, mRNA abnormalities are usually detected in three main areas: aberrant expression, aberrant splicing, and AEI. Under the classical sequencing analysis scheme, abnormal expression was identified through bioinformatics analysis of OMIM gene outliers compared to other samples in the cohort, resulting in fewer than five expression outliers in most cases (Figure 2b). Splicing analysis reported a median of 10 genes per sample with novel or outlier junctions after cross‐referencing with OMIM genes (Figure 2c). AEI analysis was limited to heterozygous sites of highly suspected genes, with no positive findings in our cohort. A manual review and evaluation of retained genes and loci was conducted, focusing on genes classified as VUS in WES and phenotype‐related genes from the Human Phenotype Ontology (HPO) (Table S2).

Coverage information of splice junctions was extracted from aligned BAM files. We normalized the uniquely mapped reads at splice junctions to correct for comparability in gene expression and library size. Normalization was based on the highest shared known annotated splice junctions, and local normalization was applied to the supporting reads of each sample. For example, a novel exon–exon junction spanning two canonical exon–intron junctions was normalized based on the maximum read count supporting these canonical junctions [8]. This local normalization filtered out low‐level mapping noise and accounted for random variations in gene expression and library size across samples. Aberrant splicing events were identified by filtering based on normalized values, supporting read counts, and other criteria, specifically the following: (a) Aberrant splicing junctions contain at least one known annotated exon–exon junction, indicating splicing into an existing transcript. (b) The supporting reads for aberrant splicing are ≥ 5. (c) The normalized value is ≥ 0.05. (d) The aberrant splicing event′s normalized value is maximal within the sample and is at least twice that of the second‐highest sample. The filtered aberrant splicing fragments were manually inspected in IGV software to confirm the splicing pattern, and sashimi plots were generated using the R package ggSashimi.

### 2.8. TID Analysis

Using genome alignment BAM files and gene annotation GTF described in Gene Expression Analysis, the alternative splicing events involving all annotated transcripts were analyzed using rMATS (https://rnaseq-mats.sourceforge.io/). Significantly differential events between case and control samples were identified with the criteria of |IncLevelDifference| ≥ 0.15 and FDR ≤ 0.05. The scope of the analysis was then narrowed down to candidate genes with strong genotype–phenotype correlations, and significant differential events associated with the major transcripts in these candidate genes were further selected. Subsequently, values corresponding to these transcripts were extracted. The TID value, ratio of the isoform to the representative transcript, was calculated based on the TPM of transcripts corresponding to differential events. Finally, transcripts with TID outliers were verified by RT‐PCR and Sanger sequencing using transcript‐specific primers.

### 2.9. Read Realignment Against the MANE‐Selected Reference Transcriptome

To address potential issues where mapping sequencing reads directly to the genome may be affected by splice sites between exons, reads were mapped to MANE transcripts using BWA (v0.7.17). Picard (v1.119) was then used for MarkDuplicates, and GATK (v3.8.1) HaplotypeCaller was utilized to call indels. To minimize false positives, case and control samples were processed in parallel, and only indel events uniquely observed in case samples but absent in controls were retained. Finally, the sequences of these specific indels were mapped back to the reference genome, thereby further ensuring the elimination of naturally occurring isoforms. Candidate indels identified through this pipeline were retained for subsequent validation.

### 2.10. Confirmation of Positive Aberrant Events

To confirm aberrant splicing events detected by RNA‐seq, PCR was performed with cDNA samples using specific primers (Table S5). The PCR products were subjected to agarose gel electrophoresis and Sanger sequencing to determine changes in pre‐mRNA splicing. DNA sequences were retrospectively verified in WES/WGS data and by Sanger sequencing of the genomic regions containing suspected variants.

The authors confirm that no artificial intelligence–generated content (AIGC) tools—including ChatGPT and other tools based on large language models (LLMs)—were used in the development of any portion of this manuscript.

## 3. Results

### 3.1. PHACs Demonstrate Transcriptomic Fidelity and Clinical Utility for RNA‐Seq Applications

The activation of T cells with PHA, a routine method for karyotype testing in genetic laboratories [27], provides a practical and efficient solution. This approach requires only a minimal blood volume to culture rapidly proliferating T cells, thereby reducing sample turnaround‐time constraints. Furthermore, the 3–5‐day cultivation period allows controlled modulation of gene expression, potentially providing supportive evidence for pathogenic variant annotation. Consequently, the clinical adoption of PHACs in genetic counseling workflows is promising. However, their suitability for RNA‐seq requires systematic evaluation. To address this, we performed comparative RNA‐seq analyses of PHACs and PBMCs. Flow cytometry of CD45^+^ lymphocytes revealed that PHACs exhibited a marked reduction in CD19^+^ B cells (1.2% vs. 9.0%) and a striking enrichment of CD3^+^ T lymphocytes (77.0% vs. 32.0%), predominantly driven by elevated CD8^+^ T cell subsets (Figure S1A). Principal component analysis (PCA) of transcriptomes demonstrated enhanced homogeneity in PHACs, with tighter clustering in principal component space compared with PBMCs (Figure S1B). Notably, this consistency persisted regardless of collection protocol (on‐site vs. mailed), underscoring PHACs′ robustness to preanalytical variability.

Comparative quality assessment demonstrated that, while both sample types yielded comparable metrics, PHACs exhibited superior performance in key RNA quality parameters, including RNA integrity (RIN/RQN: 9.8 ± 0.4 vs. 9.0 ± 1.3; *p* < 0.0001) and mapping rates (98.6*%* ± 0.4*%* vs. 98.4*%* ± 0.3*%*; *p* = 0.0088) (Figure S1C, Table S3). Transcriptome profiling identified 13,156 expressed genes (median TPM ≥ 1), of which 90% (11,909 genes) showed concordant expression between PBMCs and PHACs, whereas 1247 and 535 genes exhibited group‐specific expression patterns (Figure S1D). Analysis of overlapping genes revealed highly consistent expression profiles, with 80% demonstrating > 70% exonic coverage in splicing–junction analyses (Figure S1D). Notably, PHACs displayed significantly lower intersample expression variability (Figure S1E).

In clinical applications, both methods detected similar numbers and expression levels of OMIM‐classified genes (TPM > 1; Figure 1a). However, PHACs showed superior performance in detecting disease‐associated genes from three major categories (*p* < 0.01; Figure 1b, Table S4): hereditary cancers (69.6% vs. 57.6%), musculoskeletal disorders (54.9% vs. 50.7%), and metabolic diseases (64.6% vs. 61.6%).

Differential gene expression (DGE) analysis revealed PHAC‐specific upregulation of critical pathways, including cell cycle regulation, DNA replication, and cellular metabolism (Figure S2A,B). These pathways contained 73.6% (82/111) of clinically actionable genes from the ACMG secondary findings list [28] (Figure S2C), particularly those associated with hereditary tumor syndromes (e.g., *MLH1*, *BRCA1*, *BRCA2*, and *MSH2*) [29] and cardiovascular disorders. The enhanced detection of these clinically relevant genes, which frequently harbor pathogenic germline variants in the general population [30, 31], highlights the diagnostic utility of PHACs.

### 3.2. Undiagnostic Families in Preconception Genetic Counseling for Rare Diseases

In our cohort of undiagnosed cases undergoing WES/WGS, we implemented a three‐tier classification based on the anticipated yield differences (Figure 1c, Table S2): (1) VUS group: families in whom one or more VUSs were identified through DNA‐based testing (*n* = 7); (2) suspected‐gene/variant‐negative: cases where a particular gene is strongly suspected (or one pathogenic allele is known), but an expected second variant was not found by DNA sequencing (*n* = 10); and (3) unsolved/no‐candidate: the largest subset (*n* = 38), truly open‐ended cases with no genetic leads. These three family groups encompassed a spectrum of diverse disorders, with neurodevelopmental disorders representing the most prevalent category (22/55, 40%), predominantly within the noncandidate variant scenario (Figure 1c).

### 3.3. A Three‐Tiered Analytical Framework Incorporating TID, MANE Reference Realignment, and Pharmacological Stimulation

We established an enhanced RNA‐seq analysis pipeline with sequential analytical steps (Figure 2a).

Figure 2Enhanced RNA‐seq testing for preconception genetic counseling. (a) Flow diagram of the stepwise enhanced blood sample–based RNA‐seq testing process in a preconception genetic counseling setting. The red‐dashed box highlights the optimized steps in the process; ∗PR‐Lym: pharmacologically recultured lymphocytes. (b) Aberrant splicing events in PHACs samples. The chart illustrates the number of candidate genes at each filtration step across all samples. (c) Number of aberrantly expressed genes per sample across successive filtering stages in the dataset.

**Figure:**
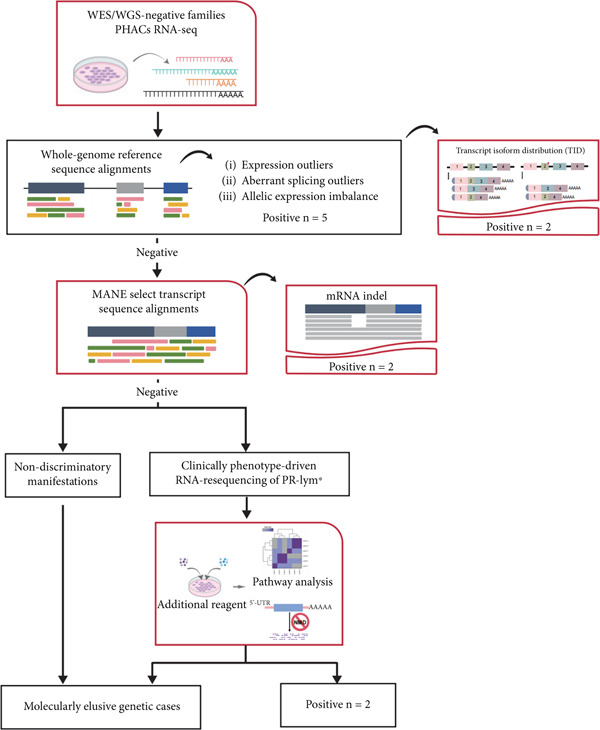
(a)

**Figure:**
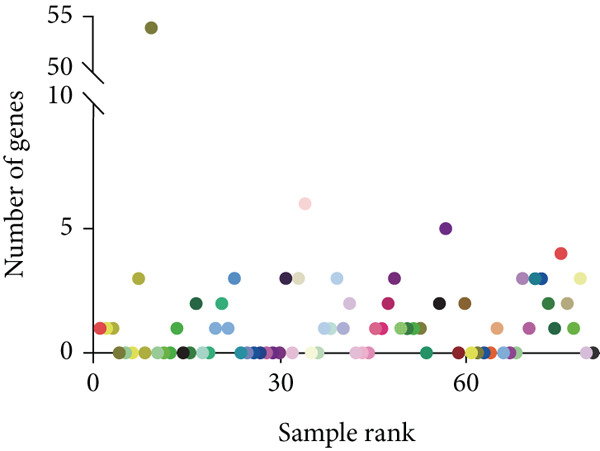
(b)

**Figure:**
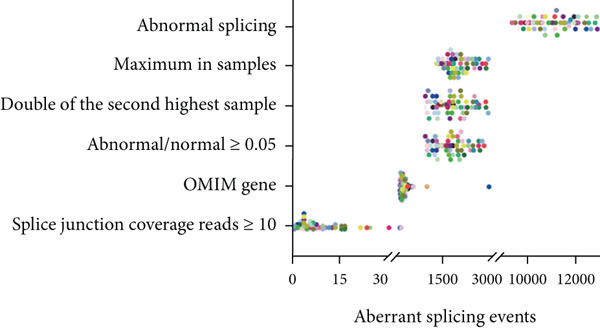
(c)

Initial transcriptome profiling followed conventional RNA‐seq processing, where aberrant expression patterns were identified by detecting OMIM gene outliers relative to cohort expression distributions (Figure 2b). This analysis revealed fewer than five expression outliers per sample in the majority of cases. Splicing variant analysis detected a median of 10.3 genes per sample containing novel or outlier splice junctions after OMIM gene filtering (Figure 2c). For allele‐specific expression (AEI) analysis, we specifically examined heterozygous sites in high‐probability candidate genes, although no pathogenic variants were identified in our cohort. Five families (Cases 11, 21, 46, 48, and 54) yielded definitive molecular diagnoses, representing a 9% diagnostic rate (Table S2).

To improve diagnostic yield in the remaining 50 unresolved cases, we developed a three‐tiered analytical augmentation strategy (Figure 2a):

1. TID analysis: Standard splicing detection methods typically identify only novel isoforms absent from reference databases as outliers (Figure 2c). However, pathogenic variants may instead cause shifts between existing isoforms—particularly when mutant alleles produce transcripts overlapping reference isoforms via mechanisms like exon skipping. TID quantifies expression ratios between all annotated transcript isoforms relative to the representative transcript (MANE‐selected), enabling statistical identification of pathological isoform redistribution patterns in two additional families not captured by conventional outlier detection.
2. MANE‐selected transcriptome realignment: To improve variant calling accuracy, we implemented secondary alignment using the MANE‐selected transcriptome as a reference. This approach circumvents alignment artifacts common in whole‐genome mapping, particularly for small indels in target genes, by providing continuous coding sequence coordinates without artifactual intron‐spanning alignments. Through this step, two positive cases were identified among the remaining 48 negative cases.
3. Pharmacological induction–based cryptic splicing detection: Aberrant isoforms may escape detection due to NMD or the intrinsically low expression under normal conditions. Cryopreserved cells from RNA‐seq negative cases with phenotypes strongly implicating specific genes (VUS and suspected‐gene/variant‐negative groups) were subjected to pharmacological perturbation (e.g., NMD inhibition with CHX or pathway‐specific agonists) to unmask cryptic splicing defects.

### 3.4. TID Unmasked Aberrant Splicing Events in Two Families

In Family 20 (Figure 3a), WES identified a heterozygous VUS (c.2019G > A, p.Leu673=) in *PKD2*, located at the last base of Exon 9. Although this variant is synonymous, SpliceAI predicted a potential impact on splicing, specifically a donor loss ([DL]: 0.83). However, routine RNA‐seq analysis did not detect any outliers. Through TID analysis, we observed an abnormally high expression ratio between the NR_156488.2 and NM_000297.4 transcripts of the *PKD2* gene in the proband and his sister (Table S6, Figure 3b). The NR_156488.2 transcript differs from NM_000297.4 only by the absence of an alternative internal Exon 9 (Figure 3d). Visualization in IGV revealed a significant reduction in read counts for Exon 9 in the proband and his sister compared with controls, while other exons remained unaffected (Figure 3c). We speculate that the c.2019G > A variant induces skipping of Exon 9 in the NM_000297.4 transcript, resulting in a spliced sequence overlapping with NR_156488.2. Notably, NR_156488.2 is classified by NCBI Gene as a noncoding transcript. The elevated NR_156488.2/NM_000297.4 ratio suggests an increased proportion of the noncoding transcript and a corresponding decrease in the functional transcript, despite no apparent change in the total *PKD2* expression (TPM). This alteration is consistent with the dosage‐sensitive pathogenic mechanism of *PKD2*.

Figure 3TID unmasked aberrant splicing events in two families. (a) Pedigree of Family 20 showing the clustering of patients with polycystic kidney disease and polycystic liver disease. (b) Expression levels of NR156488.2/NM_000297.4 in each sample. The red dot represents the proband and her cousin (affected) in Family 20, while the others denote unaffected individuals. (c) Sashimi plot of the splice junction in the *PKD2* gene in Family 20, showing that no aberrant junctions were detected. RNA coverage is indicated as depth, and the number of split reads spanning the respective intron is shown on the lines connecting the exons. The gene model of the RefSeq annotation and the aberrantly spliced exons are shown below. (d) Schematic representation of *PKD2* transcripts NR156488.2 and NM_000297.4, with arrows indicating the differences in Exon 9. (e) Pedigree of Family 47. (f) Expression levels of ENST00000637667/ENST00000249806 in each sample. The red dot represents the proband and his father in Family 47. (g) Sashimi plot of the splice junction in the *CLN6* gene in Family 47, showing no aberrant junctions. (h) Schematic representation of *CLN6* transcripts ENST00000637667 and ENST00000249806, with arrows indicating the differences in Exon 3 and Exon 9. (i) RT‐PCR and Sanger sequencing confirmed the decreased representation of transcript ENST00000249806, corresponding to longer PCR products containing Exon 3, in the proband and his father.

**Figure:**
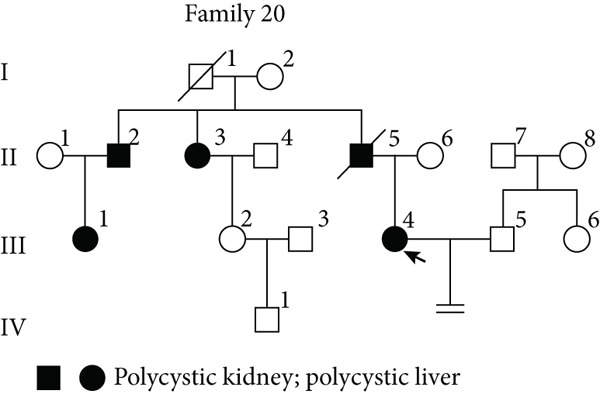
(a)

**Figure:**
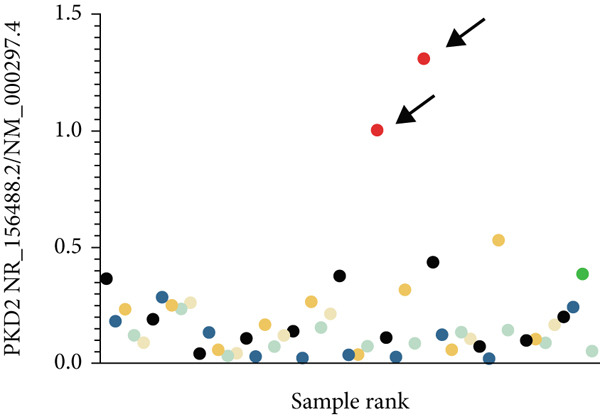
(b)

**Figure:**
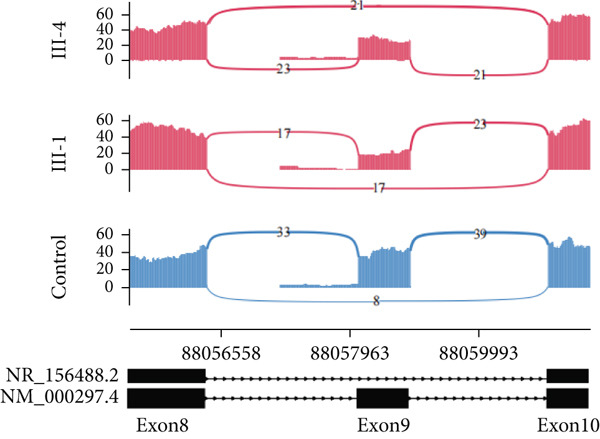
(c)

**Figure:**
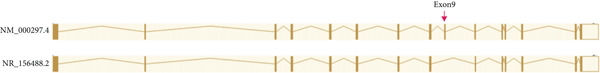
(d)

**Figure:**
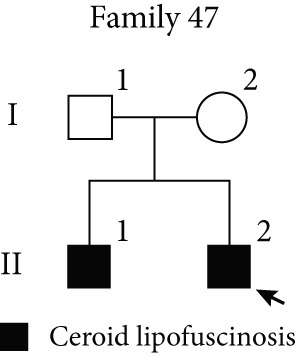
(e)

**Figure:**
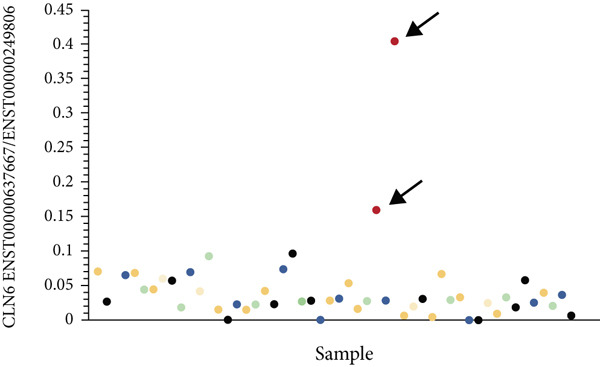
(f)

**Figure:**
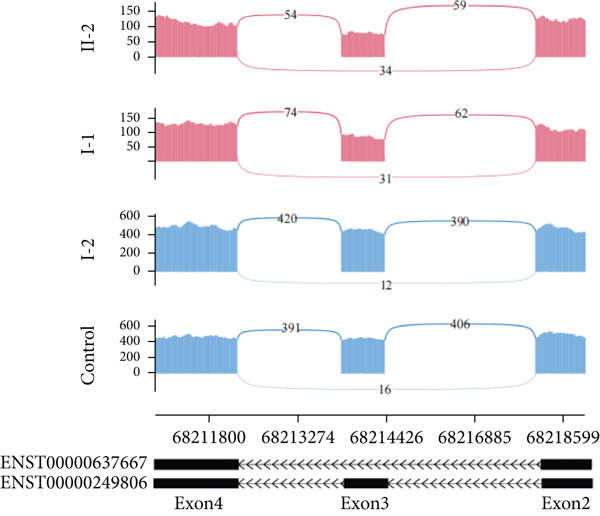
(g)

**Figure:**
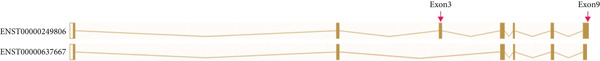
(h)

**Figure:**
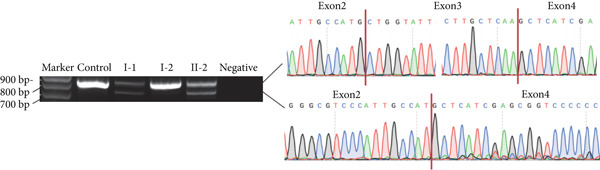
(i)

In Family 47 (Figure 3e), WES identified compound heterozygous variants in the *CLN6* gene in the proband: a maternally inherited likely pathogenic (LP) variant, c.826 T > G (p.Trp276Gly), and a paternally inherited VUS, c.297+3A > G. SpliceAI predicted that the c.297+3A > G variant would have no significant impact on splicing (score < 0.2), and routine RNA‐seq analysis did not detect any outliers. Further investigation using TID analysis revealed an abnormally high expression ratio between the ENST00000637667 transcript (no NM reference recorded) and the canonical transcript ENST00000249806 (NM_017882.3) of the *CLN6* gene in both the proband and his father (Table S6, Figure 3f). Compared with ENST00000249806, transcript ENST00000637667 lacks Exon 3 and 81 base pairs at the 3 ^′^‐end of Exon 7 (Figure 3h). Visualization in IGV confirmed a slight reduction in the signal for Exon 3 of the *CLN6* gene in the proband and his father (Figure 3g). We designed primers spanning Exon 2 and the terminal sequence of Exon 7 to specifically amplify the ENST00000249806 transcript. Using RT‐PCR and Sanger sequencing, we confirmed Exon 3 skipping in the ENST00000249806 transcript. We hypothesize that the c.297+3A > G variant induces Exon 3 skipping in the major transcript (Figure 3i). This Exon 3 skipping is predicted to result in a 33‐amino acid deletion in the *CLN6* protein, disrupting its transmembrane domain. According to ACMG guidelines, this finding provides PS3‐level evidence, thereby upgrading the c.297+3A > G variant from a VUS to LP.

### 3.5. Two Hidden Aberrant Splicing Events Uncovered Through MANE‐Selected Transcriptome Realignment

In Family 17 (Figure 4a), WES trio analysis identified a de novo heterozygous VUS in *KAT6A* (NM_006766.5: c.2436+5G > C) in the proband (II‐1), a female presenting with hemiplegia, intellectual disability, and dental anomalies. In silico prediction using SpliceAI indicated significant splicing disruption (DL score = 0.78). However, conventional genome‐aligned RNA‐seq analysis and IGV visualization failed to detect any aberrant splicing (Figure 4b). MANE transcriptome realignment revealed a previously undetected 21 bp in‐frame deletion (r.2416_2436del21) (Figure 4c), subsequently confirmed by RT‐PCR and Sanger sequencing. This finding provides PM4 evidence supporting pathogenicity, although the variant remains classified as a VUS under current ACMG guidelines.

Figure 4Two hidden aberrant splicing events revealed by MANE‐selected transcriptome realignment. (a) Pedigree of Family 17. (b) Sashimi plot of the splice junction in the *KAT6A* gene in Family 17, showing no aberrant junctions when aligned to the genome. (c) Alignment of the major reference transcript of the *KAT6A* gene (NM_006766.5) from the Ensembl and RefSeq databases with the MANE annotation in Family 17, showing a 21‐bp deletion. (d) Pedigree of Family 55. (e) Sashimi plot of the splice junction in the *GATAD2B* gene in Family 55, showing no aberrant junctions when aligned to the genome. (f) Alignment of the major reference transcript of the *GATAD2B* gene (NM_020699.4) from the Ensembl and RefSeq databases with the MANE annotation in Family 55, showing a 49‐bp deletion.

**Figure:**
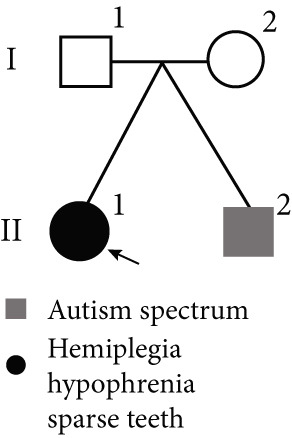
(a)

**Figure:**
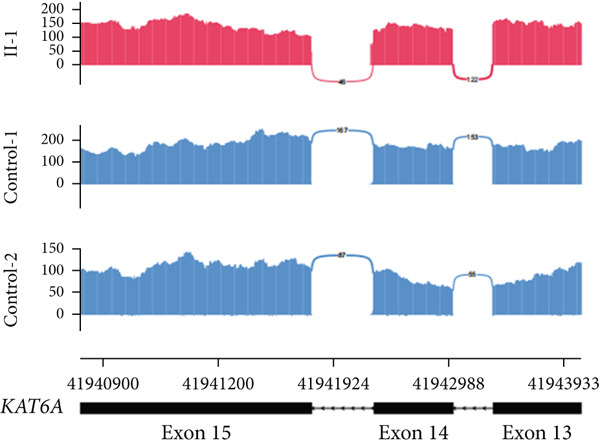
(b)

**Figure:**
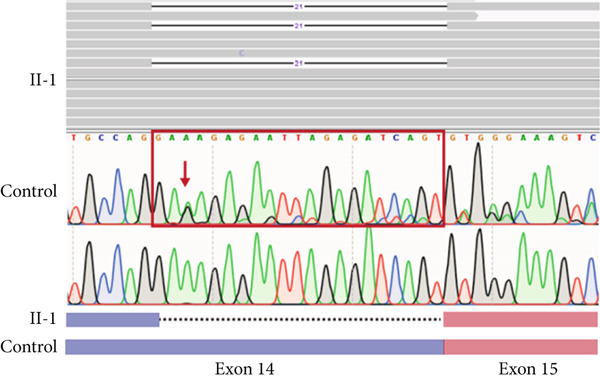
(c)

**Figure:**
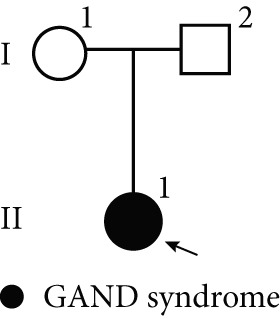
(d)

**Figure:**
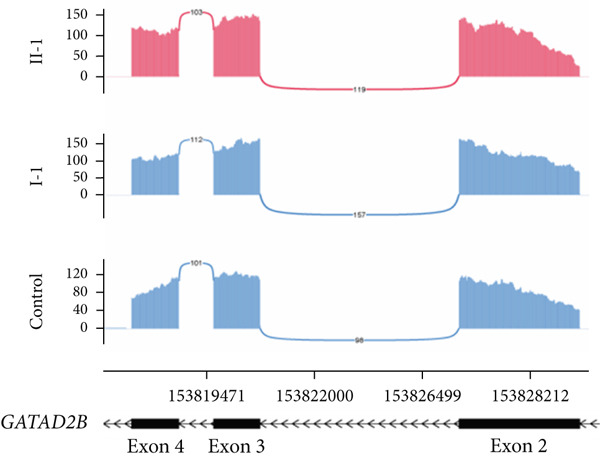
(e)

**Figure:**
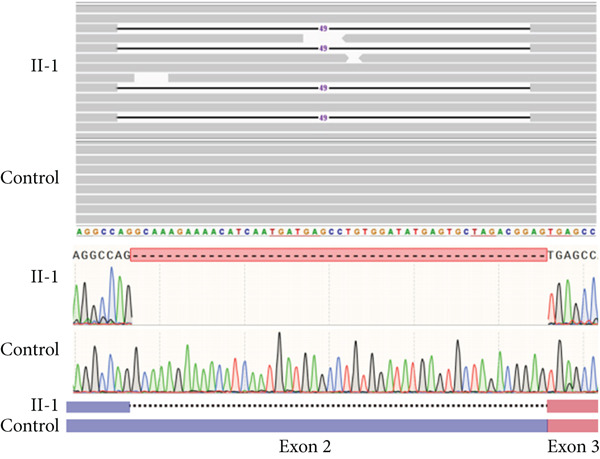
(f)

In Family 55, WES trio analysis identified a de novo synonymous variant in *GATAD2B* (NM_020699.4: c.288C > T, p.Gly96=) in a proband presenting with global developmental delay. Located 48 bp upstream of the 3 ^′^‐end of Exon 2, SpliceAI predicted this variant would strongly disrupt splicing (acceptor gain = 0.98; DL = 0.71). Initial genome‐aligned analysis and IGV inspection showed no abnormalities (Figure 4e). MANE transcriptome realignment uncovered a 49 bp deletion (r.287_335del49), predicted to cause a frameshift (p.Gly96Valfs∗8) (Figure 4f). We propose that this variant activates a cryptic splice acceptor site (c.285_286AG) within Exon 2, leading to the excision of downstream sequences. This molecular mechanism explains the predicted protein truncation.

These findings underscore the utility of MANE reference alignment in uncovering cryptic splicing events—particularly small insertions or deletions at exon boundaries that may be missed during genome‐based alignment—and refining variant interpretation in complex genetic disorders.

### 3.6. Pharmacological Induction–Based Cryptic Splicing Detection for Family 1 and Family 15

NMD represents a critical biological mechanism that must be rigorously accounted for in mRNA analysis, particularly in the context of disease‐associated transcriptomic profiling [32, 33]. NMD may degrade aberrantly spliced transcripts, leading to false negatives. CHX can indirectly block NMD activity by inhibiting translation, thereby enabling aberrant transcripts originally cleared by the NMD pathway to be retained and detected. In our cohort, a pediatric patient (Family 15) exhibiting clinical manifestations consistent with Krabbe disease underwent a comprehensive genetic evaluation (Figure 5a). Initial WES trio identified a maternally inherited pathogenic variant, c.489G > A (p.Trp163∗), within the *GALC* gene (NM_000153.3), while the second variant remained undetected. However, subsequent mRNA analysis employing the aforementioned workflow failed to yield conclusive findings. To address this, we resuscitated cryopreserved patient‐derived PHACs and implemented CHX treatment to pharmacologically inhibit NMD activity [34, 35]. This intervention enabled the detection of a previously obscured 37 bp insertion between Exons 13 and 14 of the *GALC* gene (r.1489_1490ins[1490‐453_1490‐489]) (Figure 5b,c). Genomic validation through targeted amplification of Intron 13 sequences revealed a deep intronic variant, c.1490‐452A > G (Figure 5d). Computational analysis using SpliceAI predicted significant splicing disruption (AG: 0.66; DG: 0.53), with the variant creating a novel splice donor site adjacent to a pseudoexon. This prediction is consistent with the observed RNA changes. This pseudoexon insertion causes a predicted protein truncation (p.Tyr498Aspfs∗5).

Figure 5Pharmacological induction–based cryptic splicing detection for two families. (a) Pedigree of Family 15. (b) Sashimi plot showing an aberrant splice junction in the *GALC* gene detected in PHACs treated with CHX. After CHX induction, a distinct enrichment of aberrant junctions (arrows) was observed. (c) Sanger sequencing confirmed a 37‐bp insertion between Exons 13 and 14 in *GALC* mRNA from Family 15. (d) Sanger sequencing of gDNA identified the *GALC* c.1490‐452A > G intron variants responsible for the splicing detected in Family 15. (e) Pedigree of Family 1, which includes a child with clinically ambiguous Fanconi anemia. (f) Sashimi plot showing an aberrant splice junction in the *FANCA* gene. An additional jump was observed in the patient and his father (red). The RNA coverage is indicated as depth; the number of split reads spanning the respective intron is indicated on the connecting lines between the exons. (g) Sanger sequencing confirmed the 85‐bp insertion between Exons 30 and 31 of *FANCA* mRNA in Family 1. (h) Sanger sequencing of gDNA localized the *FANCA* c.2982‐192A > G variant responsible for the aberrant transcript in Family 1. (i) DEGs between patient and control groups under PHAC or PHAC‐MMC culture conditions were intersected with the DEGs of tumor cells with normal and defective FA pathways, and KEGG analysis was performed. The dot plot shows that after adding MMC, the changes in the patient transcriptome were more similar to those in FA‐deficient tumor cells. Fanconi, FA‐deficient tumor cells; PHACs, phytohemagglutinin‐activated peripheral blood cells; PHA‐MMC, PHACs treated with mitomycin for 16–18 h.

**Figure:**
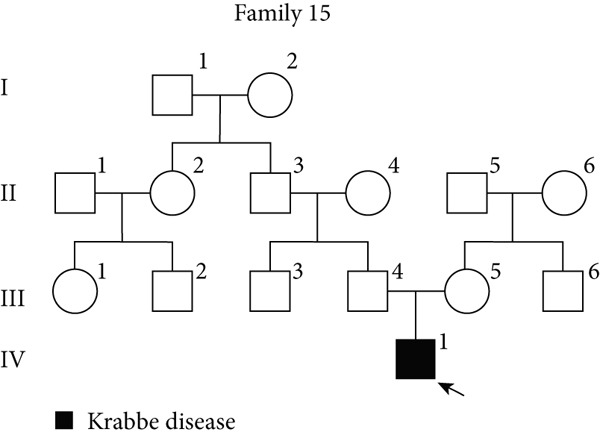
(a)

**Figure:**
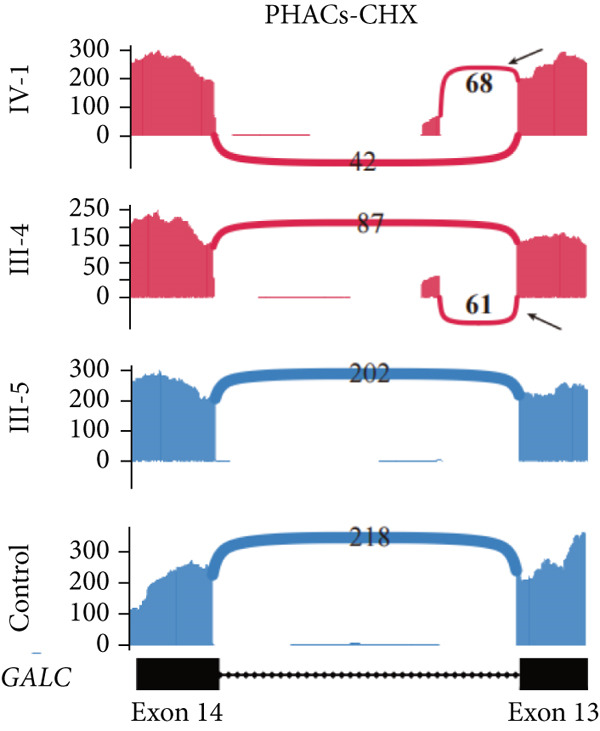
(b)

**Figure:**
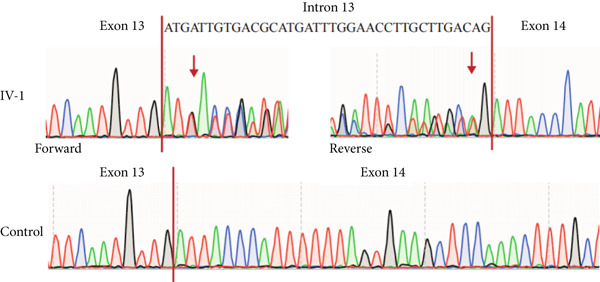
(c)

**Figure:**
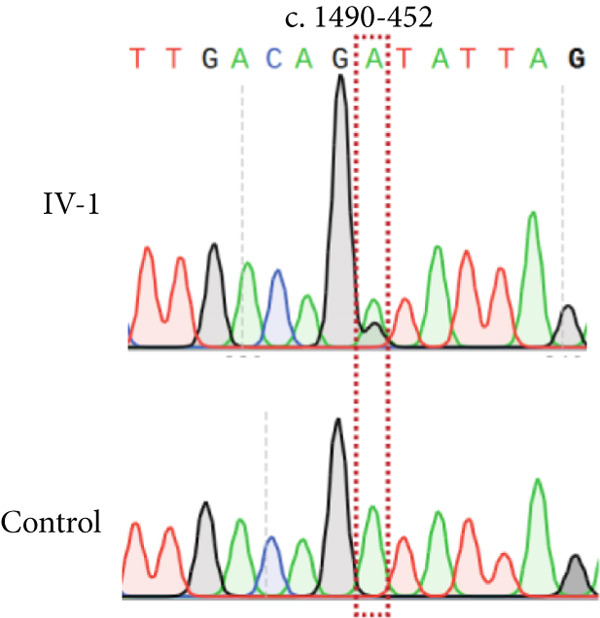
(d)

**Figure:**
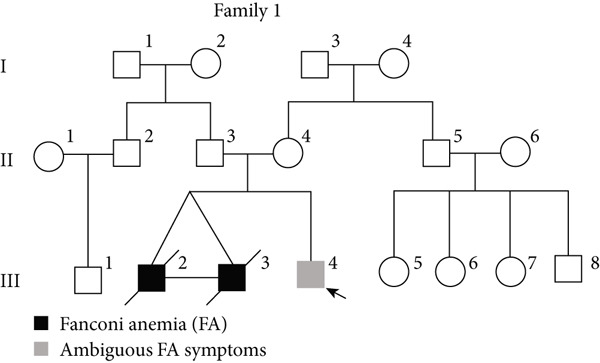
(e)

**Figure:**
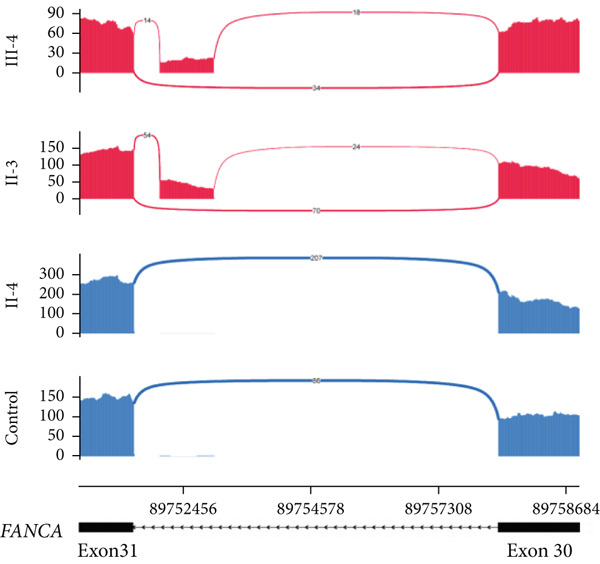
(f)

**Figure:**
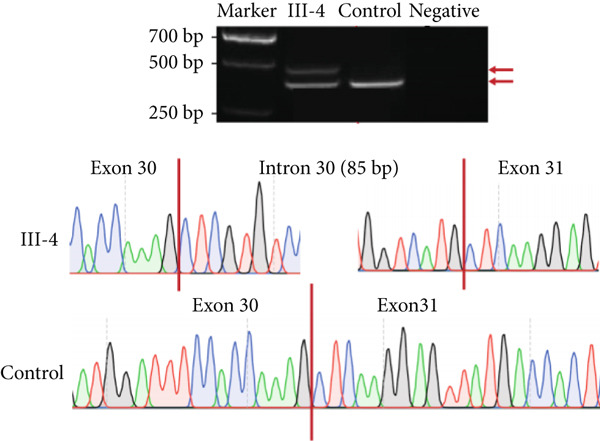
(g)

**Figure:**
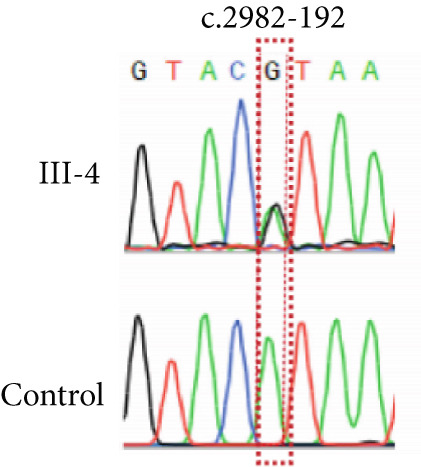
(h)

**Figure:**
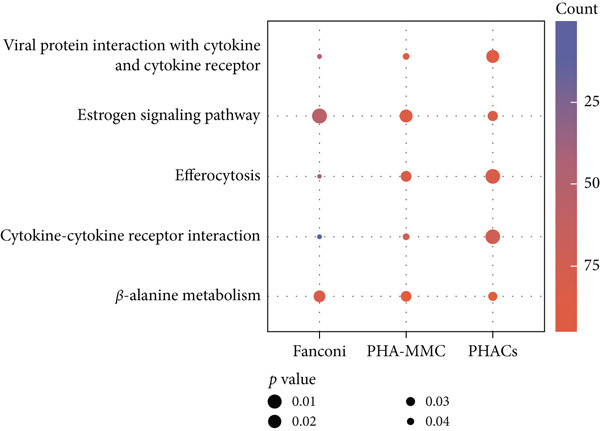
(i)

PHACs allow 3–5 days of cultivation, offering a window to modulate gene expression and uncover evidence supporting pathogenic variant annotation. Fanconi anemia (FA)—a recessive disorder caused by defective DNA interstrand crosslink repair—results in chromosomal instability [36]. In Family 1, FA‐diagnosed twins died before their parents′ hospital visit. The younger male sibling (III‐4, Figure 5e) presented with ambiguous clinical features, including multiple café‐au‐lait spots, strabismus, and a unilateral inguinal hernia. Notably, his growth parameters and hematologic indices were normal, and chromosomal breakage tests showed no significant differences versus controls. WES trio detected only a maternal heterozygous *FANCA* pathogenic variant, c.367C > T (p.Gln123Ter) in the third child. RNA‐seq revealed an aberrant pseudoexon between Exons 30 and 31 (r.2981_2982ins2982‐197_2982‐281) in FANCA in both the child and father (Figure 5f,g). This pseudoexon insertion causes a predicted protein truncation (p.Arg997Cysfs∗21), impairing FANCA function. Whole‐Intron 30 sequencing of gDNA identified a heterozygous c.2982‐192A > G variant in both individuals (Figure 5h). This deep intronic variant, predicted by SpliceAI to disrupt splicing, activates a cryptic donor site (DG = 0.5). However, his phenotype did not fully align with FA clinical criteria. To bridge this genotype–phenotype gap, we revived PHACs from the child and 12 nonhematological disease controls, stimulating them with MMC, a DNA crosslinking agent. DGE analysis between the child and controls in PHAC and PHAC‐MMC samples (Figure 5i) was compared with DGE profiles from FA pathway–deficient and nondeficient tumors [37]. MMC‐stimulated samples from the child exhibited a DGE pattern consistent with FA‐deficient tumors, a phenomenon absent in unstimulated samples. This suggests that, despite the absence of overt chromosomal breakage, the child′s transcriptome exhibits abnormalities under MMC stress, providing functional validation of the genetic findings. Based on the combined evidence, the *FANCA* variant c.2982‐192A > G was classified as LP.

### 3.7. Diagnostic Performance of the Enhanced RNA‐Seq Pipeline in Three Groups

**Figure 6:**
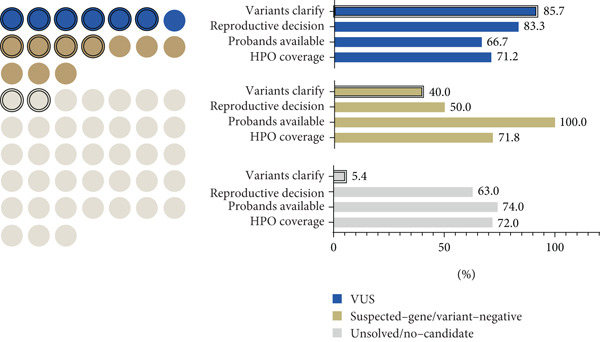
Diagnostic performance of the enhanced RNA‐seq pipeline in three groups. Schematic diagram of RNA‐seq detection across three scenarios, with edge lines highlighting cases in which effective information was obtained.

VUS group: In seven families with VUSs, RNA‐seq detected abnormal mRNA alterations in five cases (71%). Conventional analysis identified an aberrant splicing event in *C1QBP* in Family 11. Through supplemental analyses using TID and MANE‐selected transcriptome realignment, we additionally confirmed mRNA alterations in Family 20 (*PKD2*), Family 55 (*GATAD2B*), Family 17 (*KAT6A*), and Family 47 (*CLN6*). No mRNA abnormalities were detected in Families 18 and 40 after whole‐gene RNA‐seq assessment, leading to benign reclassification of *SLC26A4* (c.1341+2dupT) and *DCAF13* (c.379‐7 T > A) variants (Figure 6).

Suspected‐gene/variant‐negative group: Diagnostic yield reached 40% (4/10 families). Conventional analysis detected an *ERCC8* pathogenic variant, c.276_399del, in Family 21 and an *HGSNAT* pathogenic variant, c.372_493del, in Family 48. Pharmacological induction coupled with RNA‐seq detected a cryptic *GAL*C splicing variant c.1490‐452A > G in Family 15 and supported the pathogenicity of *FANCA* variant c.2982‐192A > G in Family 1.

Unsolved/no‐candidate group: Definitive diagnoses were achieved in 2/38 families (5.3%). *NAA15* deep‐intronic deletion (c.1014+1074_1014+1099del26; Family 46) and *GLUL* promoter variant (c.‐13‐2A > G; Family 54) were both identified through the conventional analysis pipeline.

Among the 11 RNA‐seq‐positive cases (Table 1), 10 families ultimately received a definitive genetic diagnosis. In subsequent preconception counseling, eight opted for PGT for monogenic disorders (PGT‐M), one chose prenatal diagnosis, and one selected artificial insemination by donor sperm. In another family with positive RNA alterations, but whose genetic variant remained classified as a VUS, the couple opted solely for PGT for aneuploidy (PGT‐A) due to the presence of a de novo variant and advanced maternal age (Table S2).

**Table 1:** Overview of 11 RNA‐seq‐positive cases.

**No.**	**Clinical classification**	**Phenotype**	**Results of RNA-seq**			
			**Aberrant event**	**Gene**	**Effect on mRNA**	**gDNA alteration**
1	Suspected‐gene/variant‐negative	Fanconi anemia	Splicing outlier, pharmacological induction	*FANCA* (NM_000135.4)	r.2981_2982ins2982‐197_2982‐281	c.2982‐192A > G
11	VUS	Congenital heart disease	Splicing outlier	*C1QBP* (NM_001212.4)	Exon 4 skipping	c.576+2T > A
15	Suspected‐gene/variant‐negative	Krabbe disease	Splicing outlier, pharmacological induction	*GALC* (NM_000153.3)	r.1489_1490ins1490‐453_1490‐489	c.1490‐452A > G
17	VUS	Global developmental delay, ID, appendicular hypotonia	MANE‐selected transcriptome realignment	*KAT6A* (NM_006766.5)	r.2416_2436del21	c.2436+5G > C
20	VUS	Polycystic kidney, polycystic liver	TID	*PKD2* (NM_000297.4)	Exon 9 skipping	c.2019G > A
21	Suspected‐gene/variant‐negative	Cockayne syndrome	Splicing outlier, expression outlier	*ERCC8* (NM_000082.4)	Exon 4 skipping	c.276_399del (Exon 4del)
46	Unsolved/no‐candidate	Developmental delay and ID	Splicing outlier, expression outlier	*NAA15* (NM_057175.5)	r.1014_1015ins[r.1014+1102_1014+1199]	c.1014+1074_1014+1099del26
47	VUS	Ceroid lipofuscinosis	TID	*CLN6* (NM_017882.3)	Exon 3 skipping	c.297+3A > G
48	Suspected‐gene/variant‐negative	Mental retardation	Splicing outlier	*HGSNAT* (NM_152419.3)	Exon 3‐4 skipping	c.372_493del (Exon 4del)
54	Unsolved/no‐candidate	Seizures, ID	Splicing outlier	*GLUL* (NM_001033044.4)	r.‐13_13del26	c.‐13‐2A > G
55	VUS	GDD, ID	MANE‐selected transcriptome realignment	*GATAD2B* (NM_020699.4)	r.287_335del49	c.288C > T(p.Gly96=)

## 4. Discussion

We herein present the first reported rare disease RNA‐seq diagnostic cohort utilizing PHAC specimens. Our findings establish PHACs as a robust alternative to conventional PBMCs for clinical transcriptomic applications, offering three key advantages: (1) a stable, T‐cell‐dominant transcriptional profile, (2) comparable gene detection with improved signal‐to‐noise ratios, and (3) remarkable resistance to transportation stress. Additionally, variants in ACMG secondary finding–related genes have attracted increasing attention in reproductive medicine because of their high frequency of pathogenic germline variants in the general population [29–31] and their critical role in risk stratification and personalized medical management of asymptomatic individuals [28] (Figure S2C). The advantage of PHACs in detecting secondary finding genes provides new possibilities for the future application of RNA‐seq in population screening. Although PHACs demonstrate the advantages outlined above, the detection rates of the two sample types were not directly compared using paired analyses. As shown in Figure 1a and Table S4, PHACs and PBMCs exhibit largely overlapping gene coverage, supporting the use of PHACs as an alternative material for clinical sample preparation.

However, it must be acknowledged that genes with truly tissue‐specific expression (e.g., brain‐ or muscle‐specific genes, e.g., DMD [Duchenne muscular dystrophy], NEB [nemaline myopathy], and L1CAM [hereditary spastic paraplegia]) will remain undetectable from any blood‐based RNA‐seq, suggesting that RNA‐seq from PHACs may be underpowered to detect relevant transcriptional aberrations in certain genes [8]. In practice, this means that a negative RNA result in some cases could represent a false negative simply due to a lack of gene expression. Various methods have been developed by other groups to induce RNA expression that is not normally present in blood cells. For example, a recent report [12] used transdifferentiation of induced neurons to boost neuron‐specific RNA expression. The majority of families in this cohort carry loss‐of‐function variants (affecting expression levels, transcripts usage, or splicing), while in a minority of rare diseases, gain‐of‐expression/function (GOE/F) mechanisms drive pathology [38, 39]. In such contexts, the PHAC method can theoretically still detect overexpression phenotypes. However, when a causal gene is constitutively expressed at maximal levels or decoupled from normal regulation, further induction may not yield measurable differences. Future work should include known GOE/F cases to benchmark the sensitivity of PHAC samples in overexpression disease scenarios.

Meanwhile, PHA stimulation can alter the gene expression profile, with some genes being artificially up‐ or downregulated by PHA (e.g., upregulating cell cycle and DNA repair pathways) (Table S4, Figure S2). This observation brings two key considerations for clinical application: (1) In practice, it is necessary to select more appropriate samples by combining the disease type and the range of suspected genes. (2) Given the intrinsic variation in RNA expression profiles across samples of different ages and sexes [40, 41], future clinical studies should continue to accumulate samples to establish sufficiently large control subgroups stratified by age and sex, thereby more effectively accounting for artifactual RNA processing induced by PHA treatment itself.

Through TID analysis, we identified two variants that escaped detection by conventional RNA‐seq analysis. In Family 47, the pathogenic *CLN6* variant triggered complete Exon 3 skipping but was missed by standard outlier‐calling pipelines because public databases catalog naturally occurring functional isoforms lacking this exon. These database‐registered isoforms differ from the representative transcript by only minor sequence variations outside Exon 3, leading to a previously unrecognized false‐negative result. This false‐negative result stems from short‐read sequencing′s inherent limitations. In Family 20, a *PKD2* variant produced documented nonfunctional transcripts, escaping detection due to the current pipelines′ inability to differentiate functional from noncoding isoforms. This diagnostic gap stems from incomplete functional annotation of transcript isoforms. Thus, developing algorithms based on isoform differences may be an important avenue for improving the diagnostic efficiency of RNA‐seq. Within this context, our pioneering TID approach provides a practical and cost‐effective solution to mitigate these false‐negative findings. The TID analysis utilizes available software and can be integrated into existing outlier‐detection pipelines. Outliers in TID have the potential to become a regular component of RNA‐seq analysis, similar to the analysis of gene expression levels and aberrant splicing, and thus hold promise for widespread application. However, due to the current limited knowledge regarding the existing transcript isoforms of most genes, the interpretation of the clinical significance of TID aberrant events requires functional validation.

The intrinsic discrepancy between the complexity of the human transcriptome and the linearity of the reference genome renders RNA‐seq read alignment highly susceptible to missing exon–exon junction anomalies. By implementing the MANE transcript alignment augmentation strategy, we successfully identified two splice anomalies. These anomalies were marked by junction‐spanning aberrations arising from short deletions at exon–intron boundaries. Despite the modest incremental detection rate (two of 48 cases), the low marginal operational cost of this step, particularly when guided by candidate gene prioritization, demonstrates its cost‐effectiveness. Hence, we propose integrating MANE‐guided alignment as a high‐yield optimization within clinical RNA‐seq pipelines.

PHACs facilitated ex vivo validation by utilizing cryopreserved lymphocytes, eliminating the need for patient recall. In this study, CHX‐treated lymphocytes were employed within an RNA‐seq diagnostic cohort, enabling the successful identification of transcriptomic anomalies in Family 15. Although NMD is a crucial component of RNA surveillance, our data indicated that CHX treatment induced a genome‐wide increase in aberrant splicing events (Figure S3), which exceeded the capacity of manual analysis. Consequently, we recommend its use only in cases where pathogenic variants or candidate genes have been preidentified. Therefore, this step should be limited to hypothesis‐driven investigations and to select cases where a pathogenic splice event is strongly suspected but not initially undetected, and the presence of alleles in the target gene cannot be definitively confirmed (e.g., Family 15). Indiscriminate pharmacological treatment of all samples is not recommended.

Moreover, in Family 1, PHAC induction by MMC combined with DGE analysis provided diagnostic evidence. DGE analysis using peripheral blood has already been integrated into clinical tumor diagnostics [42]. Our study pioneers the integration of this methodology into the rare‐disease RNA‐seq workflow, expanding the clinical utility of transcriptome analysis.

Our subgroup classification results indicate variability in RNA‐seq positive detection rates among families stratified by clinical diagnoses and DNA test results. RNA‐seq demonstrated the highest reclassification in the VUS group. In the suspected‐gene/variant‐negative group, RNA‐seq achieved a moderate diagnostic yield. Conversely, in the unsolved/no‐candidate group, characterized by broad or nonspecific phenotypes and thousands of potential genes (on average, our patients were associated with 1198 HPO genes), RNA‐seq exhibited the lowest positive detection rate. Therefore, in clinical practice, accurate phenotypic diagnosis is essential, and classifying cases into the suspected‐gene/variant‐negative category is recommended to maximize the RNA‐seq diagnostic efficacy.

In our rare disease cohort, the diagnostic yield underscores the practicality, efficiency, and scalability of this optimized RNA‐seq workflow as a comprehensive tool for complex preconception genetic counseling scenarios. However, widespread clinical implementation requires further validation in large‐scale studies. Building on clinical laboratory experience, this study is aimed at informing RNA‐seq algorithm development and initiating preliminary exploratory work in several areas for optimization. We envision that ongoing interdisciplinary collaboration among clinicians, algorithm developers, and clinical laboratory professionals will further enhance RNA‐seq diagnostic efficacy and clinical utility.

## 5. Conclusion

We present the first clinical implementation of PHACs as a biospecimen for RNA‐seq, establishing an enhanced RNA‐seq framework by integrating innovative approaches—TID analysis, MANE‐selected transcriptome realignment, and pharmacological induction–based cryptic splicing detection—into the conventional RNA‐seq analysis pipeline. The enhanced workflow achieved approximately a twofold increase in diagnostic yield compared with conventional whole‐blood RNA‐seq and effectively supported rare disease families in making informed decisions regarding subsequent pregnancies.

## Ethics Statement

The institutional ethics committees of the Reproductive Genetic Hospital of CITIC Xiangya ethically approved the study.

## Conflicts of Interest

The authors declare no conflicts of interest.

## Author Contributions

Conceptualization: Sicong Zeng; investigation: Jinlin Ren, Congling Dai, Pan Zhang, Wenjuan Xiao, Chunbo Xie, Shimin Yuan and Zixu Chen; funding acquisition: Sicong Zeng; methodology: Jinlin Ren, Congling Dai, Sicong Zeng and Fei Meng; resources: Juan Du, Qianjun Zhang, Wenbin He, Xiurong Li, Weiling Tang and Liang Hu; project administration and supervision: Juan Du, Guangxiu Lu and Ge Lin; writing original draft: Sicong Zeng, Jinlin Ren and Congling Dai contributed equally to this work.

## Funding

This study was funded by the Natural Science Foundation of Hunan Province, 10.13039/501100004735, 2023JJ30422 and 2022JJ40657; the Health Research Project of Hunan Provincial Health Commission, W20243089; and the Research Foundation of Reproductive and Genetic Hospital of CITIC‐Xiangya, YNXM‐202204, YNXM‐202002, and YNXM‐202205.

## Supporting Information

Additional supporting information can be found online in the Supporting Information section.

## Supporting information


**Supporting Information 1** Figure S1: Comparative analysis of cell population characteristics and transcriptomic profiles between PBMCs and PHACs. (A) Flow cytometry analysis of PBMCs and PHACs. Cell populations were defined as follows: CD3^+^/CD45^+^ (T cells), CD19^+^/CD45^+^ (B cells), CD3^+^/CD8^+^ (CD8^+^ T cells), and CD4^+^/CD3^+^ (CD4^+^ T cells). (B) Principal component analysis (PCA) based on gene expression of PBMC and PHAC samples. Red dots indicate samples delivered to the laboratory by mail. (C) Comparison of quality metrics between PHAC and PBMC samples. The *p* values are ≥ 0.05 based on a *t*‐test. (D) Comparison of transcription profiles between PBMC and PHAC. PHACs (purple); PBMCs (gray). The left bar shows the number of genes expressed in PBMCs and PHACs. The violin plot shows the distribution of gene expression values. The *y*‐axis represents the gene expression levels on a log_2_ scale, while the *x*‐axis represents the different sample groups, with the medians and interquartile ranges indicated in the graph. The bar chart shows the number of genes stratified by expression level, and the density plot represents the proportion of annotated junctions covered per gene. (E) Comparison of intersample expression variability between PBMCs and PHACs.

**Supporting Information 2** Figure S2: Comparative analysis of differential gene expression and pathway enrichment between PBMCs and PHACs. (A) Volcano plot comparing significantly (*p* value < 0.05) upregulated (log_2_ fold change ≥ 1, red) or downregulated (fold change ≤ 1, blue) genes between PBMCs and PHACs. (B) Kyoto Encyclopedia of Genes and Genomes (KEGG) analysis for PBMCs and PHACs, highlighting key pathways involved. (C) Analysis of secondary finding (SF) genes demonstrating enhanced analytical capability in PHACs. The TPM (transcripts per million) values for common SFs are presented for PBMCs (gray) and PHACs (purple).

**Supporting Information 3** Figure S3: Comparison of aberrant splicing events before and after CHX treatment in PHACs.

**Supporting Information 4** Table S1: All subjects and samples in the study. Table S2: Overview of all families. Table S3: Quality metrics for samples in the study. Table S4: List of genes available for transcriptome sequencing (TPM > 1) and analysis across nine systems for PBMCs and PHACs. Table S5: Primers used for RT‐PCR and Sanger sequencing. Table S6: List of TID events for Families 20 and 47.

## Data Availability

The data that support the findings of this study are available on request from the corresponding author. The data are not publicly available due to privacy or ethical restrictions.
