# Supplementary figures and images for: RNA‐Seq of Cultured Peripheral Blood Lymphocytes Improves Identification of Cryptic Splicing Defects in Rare Disease Diagnostics
Source: Hum Mutat. 2026 Jan 8;2026:9635551. doi: 10.1155/humu/9635551 (PMC12781863; doi:10.1155/humu/9635551)

A

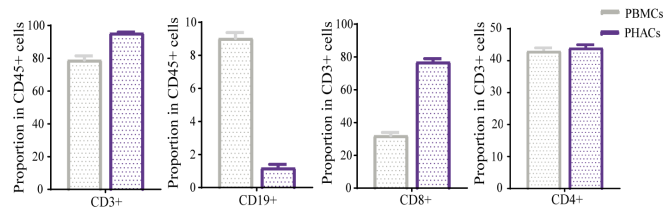

B

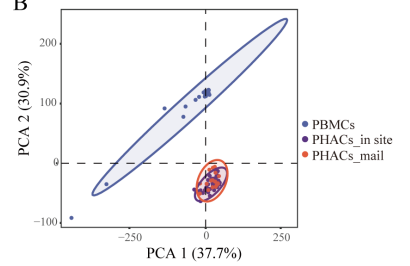

C

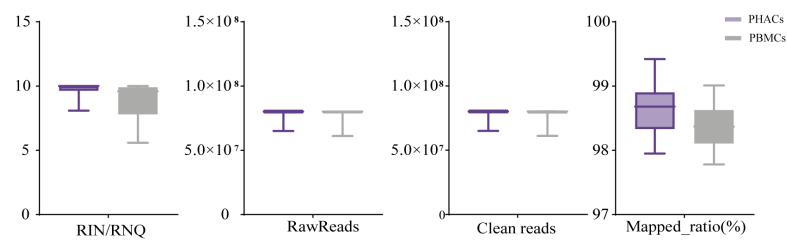

E

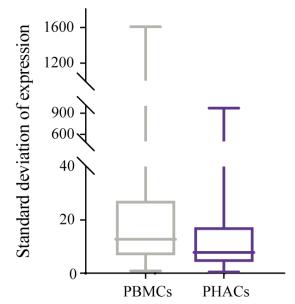

D

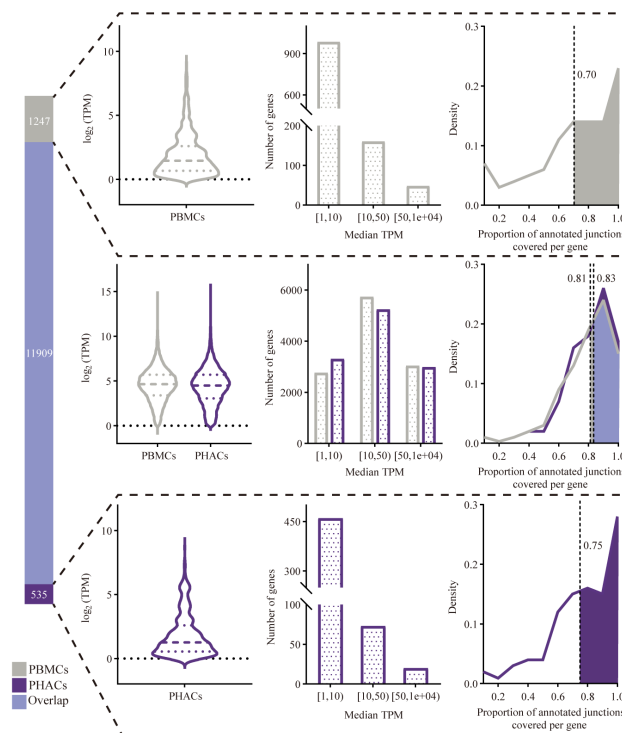

Supplement: Supplementary file 1 — Supporting Information 1 Figure S1: Comparative analysis of cell population characteristics and transcriptomic profiles between PBMCs and PHACs. (A) Flow cytometry analysis of PBMCs and PHACs. Cell populations were defined as follows: CD3+/CD45+ (T cells), CD19+/CD45+ (B cells), CD3+/CD8+ (CD8+ T cells), and CD4+/CD3+ (CD4+ T cells). (B) Principal component analysis (PCA) based on gene expression of PBMC and PHAC samples. Red dots indicate samples delivered to the laboratory by mail. (C) Comparison of quality metrics between PHAC and PBMC samples. The p values are ≥ 0.05 based on a t‐test. (D) Comparison of transcription profiles between PBMC and PHAC. PHACs (purple); PBMCs (gray). The left bar shows the number of genes expressed in PBMCs and PHACs. The violin plot shows the distribution of gene expression values. The y‐axis represents the gene expression levels on a log2 scale, while the x‐axis represents the different sample groups, with the medians and interquartile ranges indicated in the graph. The bar chart shows the number of genes stratified by expression level, and the density plot represents the proportion of annotated junctions covered per gene. (E) Comparison of intersample expression variability between PBMCs and PHACs. [file HUMU-2026-9635551-s004.pdf]

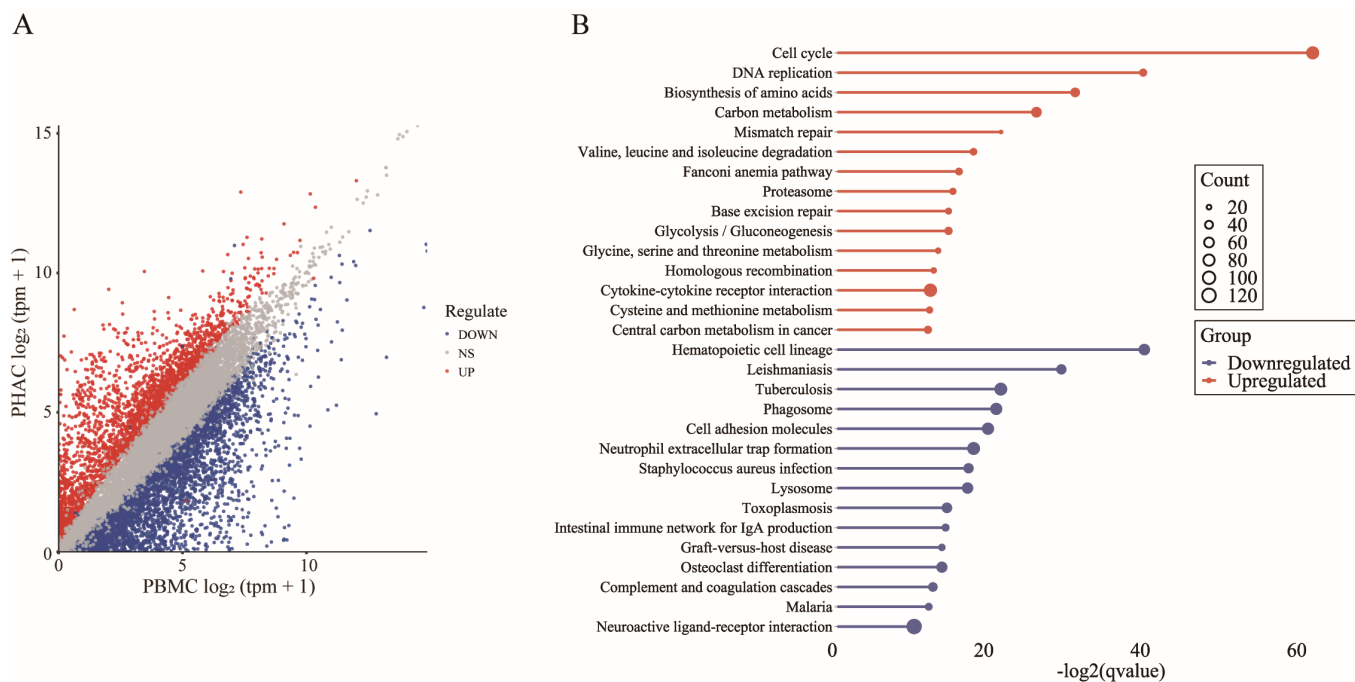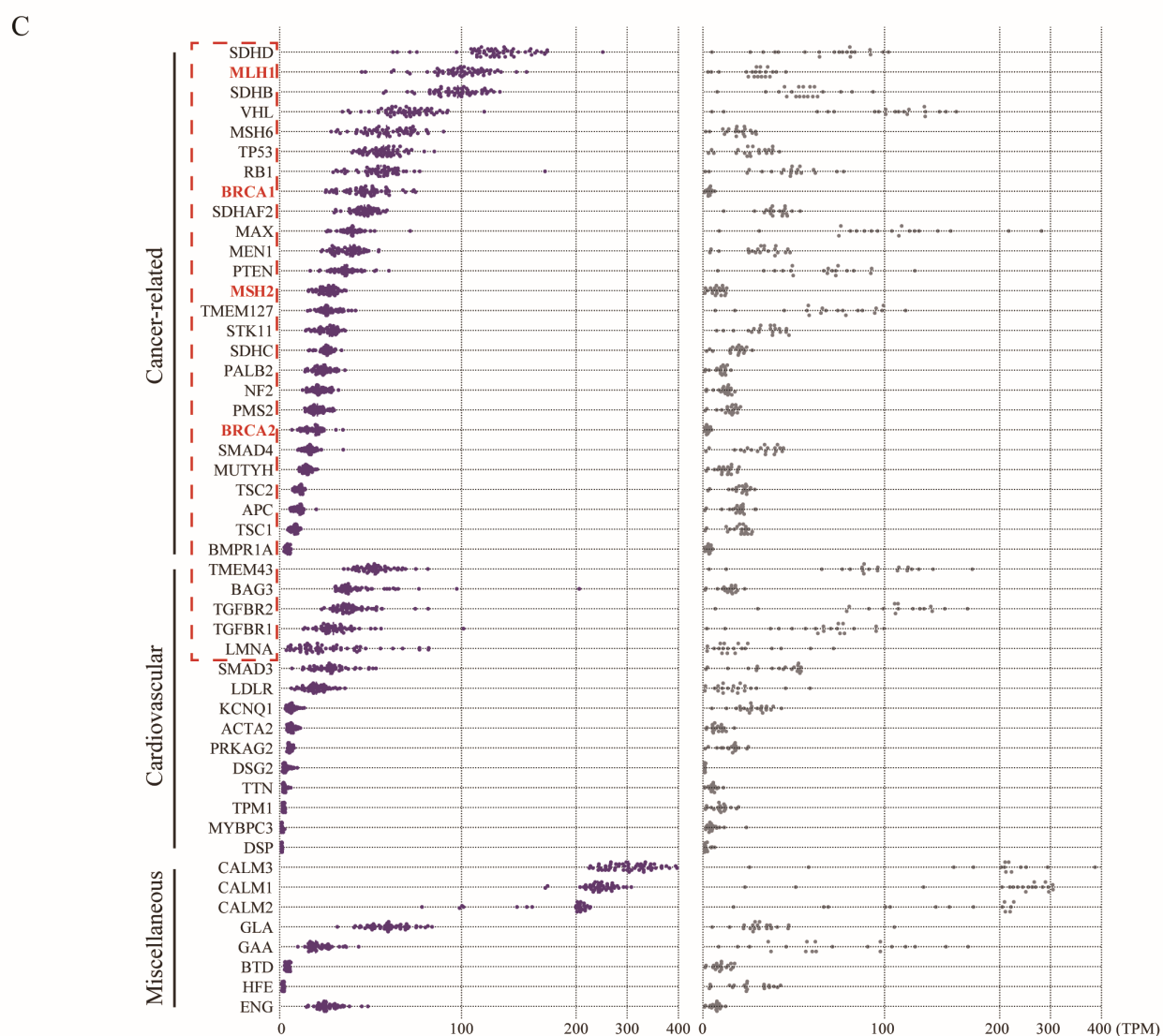

Supplement: Supplementary file 2 — Supporting Information 2 Figure S2: Comparative analysis of differential gene expression and pathway enrichment between PBMCs and PHACs. (A) Volcano plot comparing significantly (p value < 0.05) upregulated (log2 fold change ≥ 1, red) or downregulated (fold change ≤ 1, blue) genes between PBMCs and PHACs. (B) Kyoto Encyclopedia of Genes and Genomes (KEGG) analysis for PBMCs and PHACs, highlighting key pathways involved. (C) Analysis of secondary finding (SF) genes demonstrating enhanced analytical capability in PHACs. The TPM (transcripts per million) values for common SFs are presented for PBMCs (gray) and PHACs (purple). [file HUMU-2026-9635551-s003.pdf]

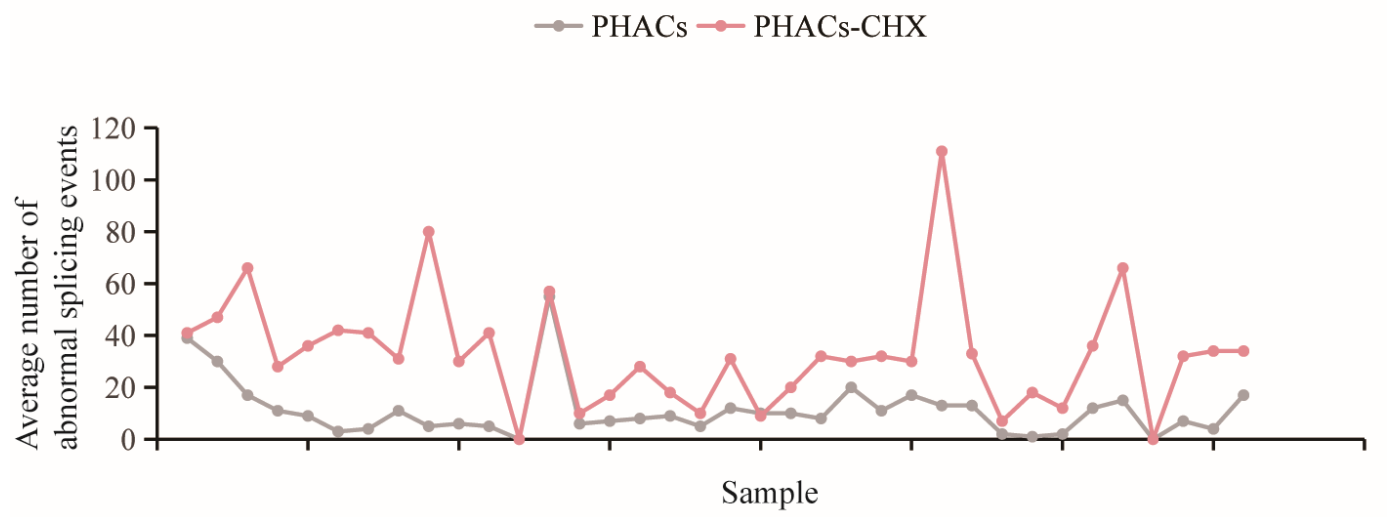

Supplement: Supplementary file 3 — Supporting Information 3 Figure S3: Comparison of aberrant splicing events before and after CHX treatment in PHACs. [file HUMU-2026-9635551-s002.pdf]
